# Acute and inherited piRNA-mediated silencing in a *rde-3* ribonucleotidyltransferase mutant

**DOI:** 10.17912/micropub.biology.000638

**Published:** 2022-09-14

**Authors:** Monika Priyadarshini, Sarah AlHarbi, Christian Frøkjær-Jensen

**Affiliations:** 1 King Abdullah University of Science and Technology (KAUST), Biological and Environmental Science and Engineering Division (BESE), KAUST Environmental Epigenetics Program (KEEP), Thuwal, 23955-6900, Saudi Arabia; 2 Current address: Department of Genetics, Stanford University School of Medicine, Stanford, CA 94305, USA

## Abstract

We recently developed a piRNA-based silencing assay (piRNAi) to study small-RNA mediated epigenetic silencing: acute gene silencing is induced by synthetic piRNAs expressed from extra-chromosomal array and transgenerational inheritance can be quantified after array loss. The assay allows inheritance assays by injecting piRNAs directly into mutant animals and targeting endogenous genes ( *e.g.* , *him-5* and *him-8* ) with obvious phenotypes (increased male frequency). Here we demonstrate the piRNAi assay by quantifying acute and inherited silencing in the ribonucleotidyltransferase *rde-3 (ne3370)* mutant. In the absence of *rde-3,* acute silencing was reduced but still detectable, whereas inherited silencing was abolished.

**Figure 1. Testing acute and inherited silencing in rde-3 mutants .**
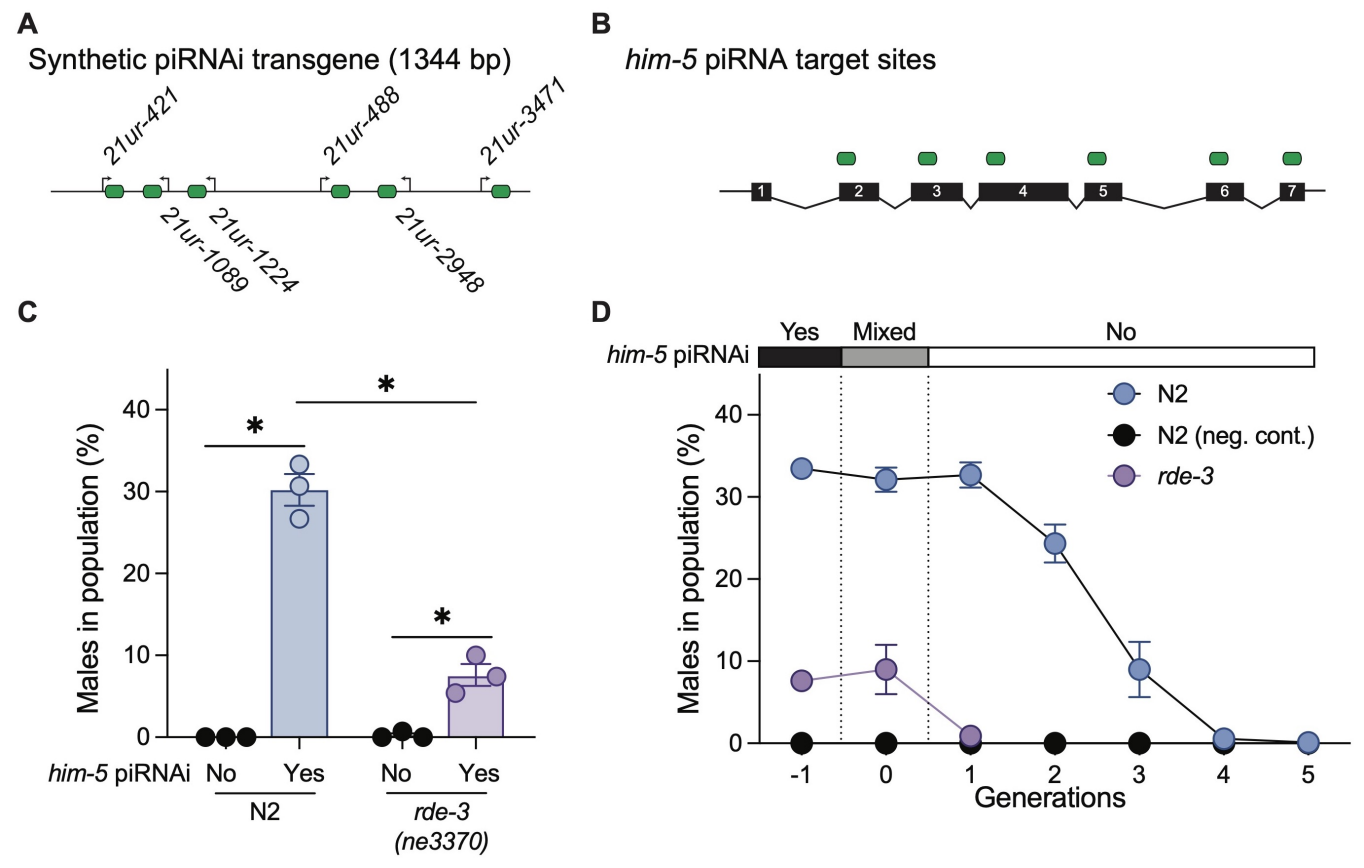
**A.** Schematic of synthetic piRNAi construct. **B** . Synthetic piRNAs target sites in *him-5* . **C.** Quantification of males frequency after piRNAi against *him-5* in N2 wild type and *rde-3(ne3370)* animals; N2: N=3, *rde-3* : N=3 ( *him-5* piRNAi and neg. control); Statistics: Mann-Whitney one-tailed test, N2 vs control (P = 0.05), *rde-3* vs control (P = 0.05), N2 vs *rde-3* (P = 0.05). **D.** Inherited *him-5* piRNAi silencing in N2 and *rde-3* animals; N = 3 all conditions. Negative control: non-targeting piRNAs. The bar above the graph indicates the presence ("yes"), mixed generation ("mixed"), and absence ("no") of a P *myo-2* :: *mCherry* marked extra-chromosomal array expressing synthetic piRNAs targeting *him-* 5. Each data point is a biologically independent transgenic line for panel **C,** and the average of three biologically independent transgenic lines for panel **D** . Error bars indicate the standard error of the mean (SEM).

## Description

*C. elegans* is a convenient model for studying small RNA-mediated inherited silencing due to the animal's short generation time (three days) and the ability to identify molecular pathways in genetic screens (Burton *et al.,* 2011; Buckley *et al.,* 2012; Spracklin *et al.,* 2017). Epigenetic silencing of an endogenous gene is often done by targeting a temperature-sensitive gain-of-function allele of *oma-1(zu405)* with dsRNA, and silencing persists for up to three generations (Alcazar *et al.,* 2008). For a visual read-out, single-copy transgenes with GFP expression in the germline (Zeiser *et al.,* 2011; Frøkjær-Jensen *et al.,* 2012; Nance and Frøkjær-Jensen, 2019) have been engineered to contain endogenous piRNA binding sites in the 3' UTR (Ashe *et al.,* 2012; Lee *et al.,* 2012; Shirayama *et al.,* 2012; Bagijn *et al.,* 2012). For transgenes, piRNA-induced silencing persists longer and sometimes indefinitely. Genetic factors required for small RNA-mediated inherited silencing have primarily been identified by crossing silenced piRNA *gfp* sensor strains into mutant genetic backgrounds (Ashe *et al.,* 2012; Lee *et al.,* 2012; Shirayama *et al.,* 2012; Luteijn *et al.,* 2012). However, introducing mutations by genetic crosses raises several concerns. First, there are several examples of mating causing changes in epigenetic inheritance. For example, the lack of transgene pairing during meiosis after a cross can lead to permanent transgene silencing via PRG-1-dependent mechanisms (Leopold *et al.,* 2015), and mating can induce multigenerational silencing inherited for over 300 generations (Devanapally *et al.,* 2021). Moreover, Dodson and Kennedy (2019) characterized a transgenerational disconnect between the genotype and phenotype (sensitivity to exogenous RNAi) of *meg-3/4* mutants for more than seven generations after a genetic cross. Second, crosses frequently require molecular genotyping, which makes it cumbersome to perform many biological replicates. Third, there are some concerns about using transgenes as a proxy for endogenous gene silencing. For example, most piRNA sensor strains include synthetic piRNA binding sites in the 3' UTR (Ashe *et al.,* 2012; Lee *et al.,* 2012; Shirayama *et al.,* 2012; Bagijn *et al.,* 2012), but endogenous genes are resistant to piRNA silencing when targeting their 3' UTRs (Priyadarshini *et al.,* 2022; Wu *et al.,* 2022). Moreover, transgene insertion site (Frøkjær-Jensen *et al.,* 2014), non-coding DNA structures (Frøkjær-Jensen *et al.,* 2016), coding sequence (Fielmich *et al.,* 2018; Aljohani *et al.,* 2020), and transgene structure (El Mouridi *et al.,* 2022) can influence epigenetic silencing. These observations suggest that transgenes may not fully recapitulate the balance between silencing foreign DNA and protecting endogenous gene expression (Frøkjær-Jensen, 2019). Finally, distinguishing between silencing initiation and maintenance phases is complicated using genetic crosses. Experiments require crossing mutant alleles to sensor strains, de-repress silencing, and outcrossing mutations to monitor *de novo* establishment of silencing (Shirayama *et al.,* 2012).

We recently developed a method called piRNA interference (piRNAi) that can efficiently silence both transgenes and endogenous genes by expressing synthetic piRNAs from arrays generated by injection (Priyadarshini *et al.,* 2022; Gajic *et al.,* 2022). Using piRNAi, we identified two endogenous targets, *him-5* and *him-8,* that inherit silencing for three and six generations, respectively (Priyadarshini *et al.,* 2022). *him-5* (Meneely *et al.,* 2012) and *him-8* (Phillips *et al.,* 2005) mutants are generally healthy but have a similar loss-of-function phenotype that is easy to score (~35% males in the population). We reasoned that piRNA-mediated silencing of *him-5* or *him-8* might be useful as a tool to directly test the role of gene mutations in initiating and maintaining inherited silencing. Here, we show that piRNAi can be used to test acute and inherited silencing in *rde-3* , a gene also known as *mut-2* (Davis *et al.,* 2022).

*rde-3* is required for Tc1 transposon silencing in the germline (Collins *et al.,* 1987) and RNA interference (RNAi) (Chen *et al.,* 2005). *In vitro* , RDE-3 has ribonucleotidyltransferase activity (Preston *et al.,* 2019) and, in *vivo* , *rde-3* is required for the addition of non-templated poly (UG) tails to the 3’ end of mRNAs targeted by RNAi and repressed transposons (Shukla *et al.,* 2020). pUGylated mRNAs are templates for RNA-dependent RNA polymerases (RdRPs), resulting in small RNA amplification and inherited silencing (Shukla *et al.,* 2020). RDE-3 is required to maintain the silencing of piRNA transgene sensors (Lee *et al.,* 2012; Shirayama *et al.,* 2012; Bagijn *et al.,* 2012). However, the role of *rde-3* in initiating silencing is unclear; re-introducing RDE-3 led to rapid re-silencing of a *gfp* :: *cdk-1* transgene, but variable and incomplete re-silencing of a *gfp* :: *csr-1* transgene (Shirayama *et al.,* 2012). Also, *rde-3* mutants are insensitive to the injection of dsRNA targeting *unc-22* but are sensitive to dsRNA expressed from transgenes (Chen *et al.,* 2005). These conflicting results could be caused by differences between transgenes, the effects of mating, or the levels of the primary silencing dsRNA. We, therefore, decided to use piRNAi to test the role of RDE-3 in the initiation and maintenance of silencing of an endogenous gene. We targeted *him-5* with six synthetic guide piRNAs (sg-piRNAs) ( **Fig. 1A-B** ) in wild-type (N2) animals and *rde-3(ne3370)* mutants. *rde-3* is a mutator strain and is relatively unhealthy, with a small brood size and infrequently produces males. To account for an elevated male frequency in the mutant population, we generated transgenic *rde-3* animals with non-targeting sg-piRNAs as a control. Targeting *him-5* with piRNAi resulted in an increased frequency of males in N2 animals but a significantly lower male frequency in *rde-3* animals (30 ± 1.9% vs 7.6 ± 1.3%, P = 0.05, mean ± SEM) ( **Fig. 1C** ). However, male frequency in *rde-3* animals was significantly increased compared to negative controls (7.6 ± 1.3% vs 0.2 ± 0.2%, P = 0.05, mean ± SEM). We tested the role of RDE-3 in maintaining silencing by losing the piRNAi trigger (the piRNAi arrays with a P *myo-2* :: *mCherry* fluorescent marker) and scoring male frequency in the following generations. In wild-type animals, the male frequency remains elevated for at least three generations after the primary piRNAs targeting *him-5* are lost ( **Fig. 1D** ), consistent with prior observations (Priyadarshini *et al.,* 2022). In contrast, we could not detect an inherited elevation of male frequency in *rde-3* mutants ( **Fig. 1D** ). The initial frequency of males was relatively low in *rde-3* animals, which limits our ability to make strong conclusions. However, our results support a model where primary piRNAs can post-transcriptionally silence a target transcript ( *him-5* mRNA) at reduced efficiency, but *rde-3* is required for small RNA amplification and transcriptional silencing. These results support the observations by Chen *et al.* (2005) that persistently high somatic expression of dsRNA targeting *unc-22* from a plasmid causes a phenotype. In contrast, a single transient injection of *in vitro* transcribed dsRNA is inefficient. Presumably, RDE-3 amplifies the primary trigger by generating *him-5* pUG RNA templates for RdRP-mediated 22G amplification; these secondary RNAs are subsequently used to set up transcriptional silencing via repressive chromatin marks deposited by the *hrde-1* dependent nuclear RNAi pathway.

More generally, we demonstrate that piRNAi can be used as a tool to directly test genetic factors required for acute and inherited silencing of endogenous genes. Elevated male frequency (induced by targeting *him-5* or *him-8* ) is easy to score in various genetic backgrounds and allows distinguishing between silencing initiation and maintenance of endogenous genes.

## Methods

Transgenesis. Transgenic animals with piRNAi extrachromosomal arrays were generated according to standard injection protocols (Mello *et al.,* 1991). The injection mix for all experiments consisted of ~15-20 ng/µl of synthetic dsDNA piRNA fragments (Twist Bioscience), 12.5 ng/µl of a plasmid encoding hygromycin resistance (pCFJ782), and 2 ng/µl of a fluorescent co-injection marker P *myo-2::* mCherry (pCFJ90). The total concentration of the injection mix was adjusted to 100 ng/µl with a 1kb DNA ladder (1 kb Plus DNA Ladder, catalog no. 10787018, Life Technologies). This mix was injected into young adult hermaphrodite animals and allowed to recover on standard NGM plates seed with OP50 bacteria. 36-48 hours post-injection, 500 µl of 4 mg/ml stock of Hygromycin solution (Gold Biotechnology, catalog no. H-270-1) was topically added to the bacterial lawn of injection plates to select for transgenic (F1) progeny. A single healthy transgenic F2 adult was picked from each plate to generate a clonal strain, and pharyngeal mCherry fluorescence was visually confirmed.

Quantification of male frequency. Quantification of male frequency was performed as previously reported by (Priyadarshini *et al.,* 2022). Briefly, six virgin L4 hermaphrodites were picked to freshly seeded NGM plates with hygromycin selection to select for the piRNAi array. The frequency of males was determined using a dissection microscope and by visual inspection of 100 adult animals on plates incubated on ice for 30 minutes to immobilize animals.

Inherited silencing assay. Six virgin L4 animals were picked to non-selective NGM plates to obtain a mixed progeny population with and without the piRNAi array. Males were not quantified in this mixed population; however, L4 animals carrying the sg-piRNAs were propagated in parallel, and their progeny were scored for males (G0). In the following generation, non-transgenic L4 animals were carefully picked from the mixed population based on the absence of pharyngeal mCherry expression (a marker for the piRNAi array). The progeny of these animals was quantified for male frequency (G1). Male frequency was quantified in all following generations by picking L4s until the male frequency was below 1%.

Data quantification and statistics . Independently generated transgenic animals were treated as biological replicates. piRNA-mediated silencing is stochastic (i.e., most strains show robust silencing, but some strains are not silenced at all), and the data do not follow a normal distribution. We performed statistical tests using one-side parametric Mann-Whitney tests to account for this.

Software . Statistical analysis was performed with GraphPad Prism (v 9.4.1), figures were generated with Adobe Illustrator (v 26.4.1), and the manuscript was written with Microsoft Word (v 16.63.1).

## Reagents

List of strains, plasmids, and piRNAi fragments used in this study.

**Strains**

N2 Standard wildtype strain (Brenner 1974)

WM286 *rde-3* ( *ne3370* ) I

**Plasmids**

pCFJ90 P *myo-2::* mCherry *::unc-54* UTR (Frøkjær-Jensen *et al.,* 2008)

pCFJ782 P *rps-0::* HygroR

**piRNAi fragments**

T288 *him-5* (six targeting piRNAs in upper-case):

cgcgcttgacgcgctagtcaactaacataaaaaaggtgaaacattgcgaggatacatagaaaaaacaatacttcgaattcatttttcaattacaaatcctgaaatgtttcactgtgttcctataagaaaacattgaaacaaaatattaagTGAGTTAGCTTTCCGGAGCTTctaattttgattttgattttgaaatcgaatttgcaaatccaattaaaaatcattttctgataattagacagttccttatcgttaattttattatatctatcgagttagaaattgcaacgaagataatgtcttccaaatactgaaaatttgaaaatatgttTCCTCACGAAAAACCTGCCTAttGccagaactcaaaatatgaaatttttatagttttgttgaaacagtaagaaaatcttgtaattactgtaaactgtttgctttttttaaagtcaacctacttcaaatctacttcaaaaattataatgtttcaaattacataactgtgtATGCAGAGAGATCAGTAGGTActgtagagcttcaatgttgataagatttattaacacagtgaaacaggtaatagttgtttgttgcaaaatcggaaatctctacatttcatatggtttttaattacaggtttgttttataaaataattgtgtgatggatattattttcagacctcatactaatctgcaaaccttcaaacaatatgtgaagtctactctgtttcactcaaccattcatttcaatttggaaaaaaatcaaagaaatgttgaaaaattttcctgtttcaacattatgacaaaaatgttatgattttaataaaaacaaTCGATCACTGTTGACAATCACttctgtttttcttagaagtgttttccggaaacgcgtaattggttttatcacaaatcgaaaacaaacaaaaatttttttaattatttctttgctagttttgtagttgaaaattcactataatcatgaataagtgagctgcccaagtaaacaaagaaaatttggcagcggccgacaactaccgggttgcccgatttatcagtggaggaTAATCCGGCACGTAGAATGTAtctaatgtgatgtacacggttttcatttaaaaacaaattgaaacagaaatgactacattttcaaattgtctatttttgctgtgtttattttgccaccaacaaTCGATGCGACCAACTGTTTTTtcaatctagtaaactcacttaatgcaattcctccagccacatatgtaaacgttgtatacatgcagaaaacggttttttggttttaatgggaacttttgacaaattgttcgaaaatcttaagctgtcccatttcagttgggtgatcgattt

T119 Control (non-targeting piRNAs):

ctcggtcaattaaagaaagaCATTTTTCATCGGATTTGCTActaaaaaataattttaaAAACGATCATATGCAAATCCAgtgaaactttattcaaaccaaaacgtttaatcagctaattgaaacattaaaaattttatgattttgttagtttttctagcaatgtcaatgcaatcaaataattttcaagtaagatgtttaatgagttatagacttttttattaaatttttgaaaaaaaaaccgatttcagatttaagtaaaattatctctgcttctgctgcattgctgcgaaacaaaaattcctttctgtgcaaagtatagtATAAACGAGGAGCACAAATGAgtgacaattagaaatctcaccgggttttctagatcatctgaaacatataattttaaaaaattgacaccttgttcaacTGTTGCACATATCACTTTTGAtcgaaacattaaatgtctcatgatttttaaagctcttttagaacagtcgCCAATCCCCTTATCCAATTTAttgaaaacaattttctagcgagatgttaaatgagtttgttgaaacagtagattttcgtgtaaacttttgaaaacaaaaattacgttttaaataaaattatatccacttcagcagtgtgcccttgaaacaaaaaagctcgatcaaaaaatttattttttgtgaatggccaccaacttttcaggcaaaattacaaaaaaacataaaatttactgtttcaaaaagttaatataattttggcagcgcatatacctacacTGAATTTTGGCAGAGGCAATTacctctttttgaaaataaag
